# Artificial Intelligence–Assisted Cancer Status Detection in Radiology Reports

**DOI:** 10.1158/2767-9764.CRC-24-0064

**Published:** 2024-04-09

**Authors:** Ankur Arya, Andrew Niederhausern, Nadia Bahadur, Neil J. Shah, Chelsea Nichols, Avijit Chatterjee, John Philip

**Affiliations:** 1 Digital, Informatics and Technology Solutions, Memorial Sloan Kettering Cancer Center, New York, New York.; 2 Department of Translational Informatics, Memorial Sloan Kettering Cancer Center, New York, New York.; 3 Clinical & Translational Research Informatics, Memorial Sloan Kettering Cancer Center, New York, New York.; 4 Department of Medicine, Memorial Sloan Kettering Cancer Center, New York, New York.

## Abstract

Cancer research is dependent on accurate and relevant information of patient's medical journey. Data in radiology reports are of extreme value but lack consistent structure for direct use in analytics. At Memorial Sloan Kettering Cancer Center (MSKCC), the radiology reports are curated using gold-standard approach of using human annotators. However, the manual process of curating large volume of retrospective data slows the pace of cancer research. Manual curation process is sensitive to volume of reports, number of data elements and nature of reports and demand appropriate skillset. In this work, we explore state of the art methods in artificial intelligence (AI) and implement end-to-end pipeline for fast and accurate annotation of radiology reports. Language models (LM) are trained using curated data by approaching curation as multiclass or multilabel classification problem. The classification tasks are to predict multiple imaging scan sites, presence of cancer and cancer status from the reports. The trained natural language processing (NLP) model classifiers achieve high weighted F_1_ score and accuracy. We propose and demonstrate the use of these models to assist in the manual curation process which results in higher accuracy and F_1_ score with lesser time and cost, thus improving efforts of cancer research.

**Significance:**

Extraction of structured data in radiology for cancer research with manual process is laborious. Using AI for extraction of data elements is achieved using NLP models’ assistance is faster and more accurate.

## Introduction

Memorial Sloan Kettering Cancer Center (MSKCC) currently has approximately 100,000 patients with genomic testing (IMPACT; ref. 1) spanning all cancer types and continues to accrue more every day. Clinicians use this genomic data for research but lack structured clinical data to analyze alongside the genomic data. Moreover, nonclinical and operation-related analytics can benefit from structured clinical data. We use a vendor called VASTA Global to manually curate unstructured paragraph text in clinical notes which includes radiology reports. Depending on the data model, we found that a patient's full cancer history can take up to 4 to 8 hours to curate. Therefore, we want to implement artificial intelligence (AI) to automate the curation of clinical notes to save time and cost. We hope to achieve a faster curation process that would allow us to accomplish more than 1 patient per curator a day to catch up to the 100,000 MSK-IMPACT cohort. In this work, we have developed natural language processing (NLP) models to automate manual curation of the PRISSMM ontology fields (2–4) – imaging scan site, presence of cancer and cancer status from radiology reports. The format and terminology of the radiology reports depend on the imaging technique (e.g., CT, MRI, PET) scanned body site and the type of cancer cohort (e.g., pancreatic, lung, breast). We leverage NLP models to assist with curation of these different radiology reports to increase the throughput and save associated cost. NLP models have achieved high accuracy and F_1_ score in radiology applications (5). Although recurrent neural network (RNN) models have shown good performance in annotation of radiology reports (6), transformer-based NLP models such as bi-directional encoder representations from transformers (BERT; ref. 7) outperform other traditional models for NLP tasks in radiology (8). Past few years have seen development of several biomedical and clinical domain adapted BERT models (9–14) and have shown very high accuracy and F_1_ score in discriminative tasks such as text classification (15–17).

In recent year, there has been advent of large language models (LLM) that are trained on much larger corpus and have ability to generate texts unlike encoder style BERT models (18–22). There are also LLMs which have been additionally trained using medical domain data (23–25). Successful application of LLMs in medical notes have achieved comparable performance as human labelers (26, 27). However, on discriminative or natural language understanding (NLU) tasks BERT models have comparable performance with LLMs (28). Also, it requires additional pretraining and fine-tuning to achieve state of the art performance levels in clinical tasks (29) which is cost and resource intensive. For our purpose we use Google's off-the-shelf BERT base uncased model with 110M trainable parameters for fine-tuning and inferencing (30).

Fine-tuning a BERT model involves training the model with labeled dataset by adding task specific output and decoding layers at the end of the model. Curated radiology reports are used for supervised fine-tuning of the model for text classification task. A separate model is used for each task as they have smaller memory footprint and high accuracy. The fine-tuned models are deployed as application programming interface (API) endpoint for fast inferencing. Model training and deployment is done on IBM's cloud pak for data (CPD) platform which is setup on premise to meet protocols of privacy and ethical guidelines. The end-to-end pipeline from data ingestion to parsing model output is tested using a held-out test data for reporting classifier model evaluation metrics. A proposed methodology to automate manual curation of radiology reports by using the model pipeline is presented. We also leverage Shapley Additive exPlanations (SHAP; ref. 31) framework to implement explainability of the model output. This work can be extended to similar NLP curation tasks in other clinical notes.

## Materials and Methods

### Data

This study was reviewed and approved by the individual site Institutional Review Boards and/or ethics committees at Memorial Sloan-Kettering Cancer Center. The IRB protocol number is 22-106. We have approximately 350,000 radiology reports which were curated by human labelers to extract fields in the PRISSMM data model (1). A nonsignificant number of curated radiology reports of these patients originate from external providers and are not considered for this study. These curated reports provide with structured dataset for various analytics to meet clinical, operational and revenue cycle management goals.

**FIGURE 1:**
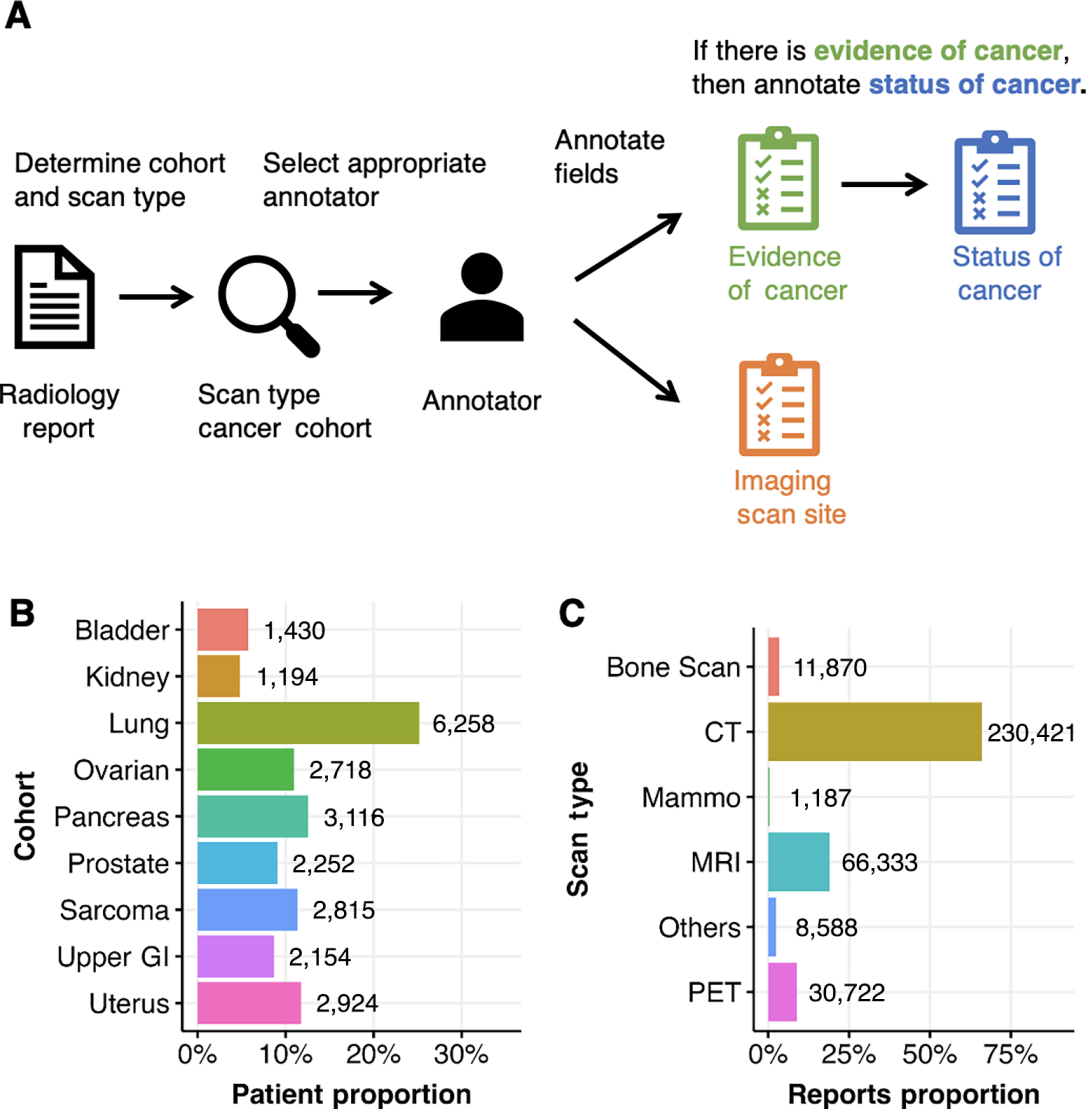
Illustration of curation workflow and data summary of curated radiology reports by cancer cohort and imaging scan technique. **A,** Illustration of manual curation workflow with dependency on imaging scan type and cancer cohort. **B,** Distribution of patients (proportion and count) with curated reports by all 9 cancer cohorts. One patient can be present in multiple cohorts. **C,** Distribution of curated reports by type of scanning technique.

The curated radiology reports are comprised of 24,000 patients grouped into 9 cohorts based on cancer type. The cancer types include bladder, lung, kidney, sarcoma, ovarian, uterine, pancreatic, prostate, and upper gastrointestinal. A patient can be present in multiple cancer cohort. The terminology and layout of the radiology report depend on the cancer cohort and imaging scan type, so these attributes of the report are important consideration for manual annotation and developing NLP models. A curation form consists of several fields, and for each of the field the curator must select either one or multiple responses depending on the field. For this work, we focus on three PRISSMM curation fields that would be the most beneficial to curation costs – imaging scan site, presence of cancer, and cancer status. The field and type of selection is used to decide which type of classifier NLP model to be used as provided in Table 1. The workflow of curation depending on the radiology report for these fields are illustrated in first panel of Fig. 1.

**TABLE 1:** List of curation fields with choices and type of selection, along with type of NLP model classifier

Field	Permissible labels	Relevant excerpt	Type of selection	Type of NLP classifier
Imaging scan site(s)	Brain/Head Spine Neck Chest Abdomen Pelvis Extremity Full body	First 500 tokens of radiology report	Multiple	Multi-label
Presence of cancer	Yes, the impression states or implies there is evidence of cancer No, the impression states or implies there is no evidence of cancer The impression is uncertain, indeterminate, or equivocal The impression does not mention cancer	Impression section	Single	Multi-class
Cancer Status	Progressing/Worsening/Enlarging Stable/No change Improving/Responding Not stated/Indeterminate Mixed	Impression section	Single	Multi-class

Imaging scan sites is used to describe what parts of the body was imaged and could be one or more selections. Imaging scan site's choice selection is interpreted from the header section of the report but also depends on the scan type, for example, PET scans are typically full body while CT and MRI can be one or more of the all the choices. This field is treated as multilabel classification task to train the NLP model classifier. The presence of cancer field, interpreted from radiologist's assessment, is a multiclass classification, where only one of the four choices is selected. Radiology reports which are curated as “Yes, the impression states or implies there is evidence of cancer” for the presence of cancer field indicate that cancer is detected in imaging and hence they are further curated for the cancer status field. The cancer status field, also interpreted from the radiologist's assessment, is a multiclass classification task where one of the 5 choices is selected by the curator. A complete list of permissible labels is listed in Table 1.

Each field is independently classified based on type of classifier and has its own independent data preparation. To prepare the data for training the model, the curated reports are first filtered to remove any missing values and identifiable incorrect curated values. For each of the classification task, 30% of data is held out for testing, rest of the data is split into training (90%) and evaluation (10%). The proportion of stratification by class or multiple label selection is kept similar across the splits.

### Model Training

**FIGURE 2:**
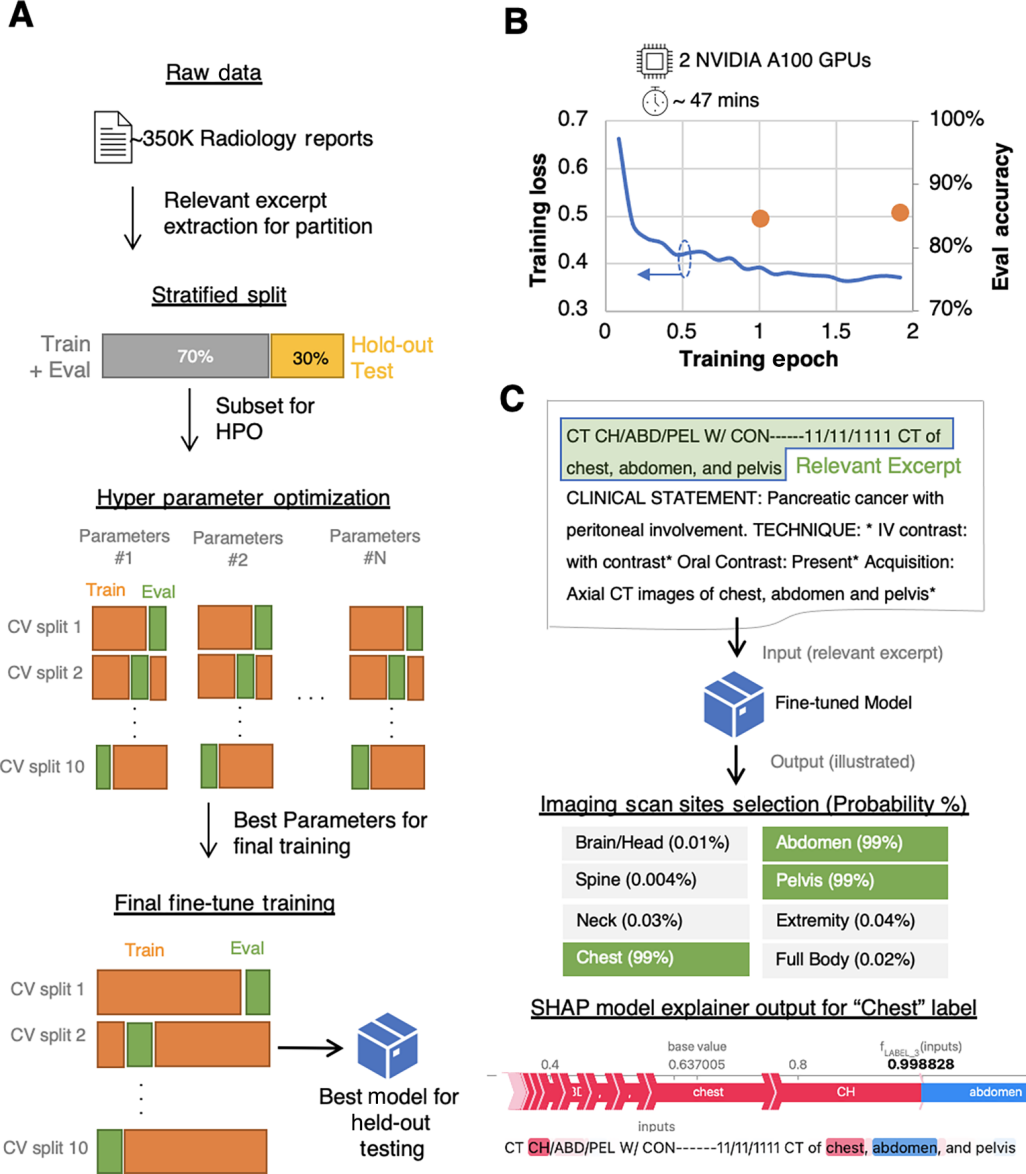
Training and inference of BERT model. **A,** Illustration of data preprocessing, data partition with balanced stratification, hyper parameter optimization (HPO) using cross validation (CV) technique and fine-tune training using CV method. **B,** Training loss curve while fine-tuning BERT model on multiclass classification of cancer status task along with exact accuracy measured after every epoch on evaluation data split. **C,** An example of inferencing of model trained on multilabel classification of imaging scan site. A radiology report excerpt (highlighted in green) is passed as input to the model, the model's output with selected label choices and corresponding label probabilities are shown. The visual from SHAP explainer model for label “Chest” is provided below model output.

Three different BERT models per task are fine-tuned using their training data with (30) as the starting checkpoint. A typical model training flow starting from data partition is illustrated in Fig. 2A. We implement 10-fold cross validation (CV) for hyperparameter optimization (HPO) of training parameters, and for model training and selection. For HPO, a small subset of the training data split is used to form CV training and evaluation splits. The parameters which lead to model evaluation with highest and least variable performance are chosen as optimal.

Due to limited input context size (512 tokens) of these models, only the relevant excerpt of the radiology reports such as impression is used in the training dataset. The relevant portion of the report is determined by the curator in annotation form and is listed in Table 1. Hence the supervised training data is a pair of task-dependent relevant excerpt and class/label(s). The fine-tuning of the model used two NVIDIA A100 graphical processing units (GPU) on IBM CPD platform installed on premise. All three models were fine-tuned with global batch sizes of 8 to 16, AdamW optimizer with constant learning rate of 2E-5, and uniform weights for computing entropy loss values. A typical training loss curve for finetuning the model for prediction of cancer status is plotted in Fig. 2B. The fine-tuned NLP model's evaluation using held-out test data are described later in results section. The example of trained model inference is shown in Fig. 2C, where the model is fine-tuned to predict multiple labels of imaging scan site. In this example, the initial part of the report is chosen as input to the model. The model outputs probabilities of selection for all the labels, and the labels with high probabilities (above threshold value of 50%) considered selected labels (refer Fig. 2C). To obtain explainability of the model output, we added a SHAP (31) explanation model in the inference pipeline. An example of the model output and SHAP visualization for prediction of multiple imaging sites is shown in bottom of Fig. 2C. The SHAP explanation model provides quantitative contribution by each token in the input text towards logits of the class or label. This feature reduces the throughput of the model pipeline and hence this feature is kept optional. The explainability feature can be used to assist human curators to reduce time and improve accuracy of the curation process, but more importantly it aids to understanding of the model prediction. The models were deployed on NVIDIA A100 GPUs using Watson machine learning accelerator framework in IBM CPD for elastic distributed inferencing (EDI). Because each model has smaller GPU memory requirement, multiple instances of same model or different models can share GPUs to maximize utilization. The deployed model is scored using an API endpoint which accepts concurrent requests with suitable policy to meet project requirements.

### Data Availability Statement

The data used in this study are proprietary to Memorial Sloan Kettering Cancer Center and are not publicly available. Data are available upon reasonable request.

## Results

Among all the curation radiology reports (*n* = 349,121) there are nine cancer cohorts. As seen in Fig. 1B, lung cancer cohort are majority with 25% patients while other cancer cohorts are within 5%–10%. The distribution of curated radiology reports by type of imaging technique is plotted in Fig. 1C. Most of the reports are from CT scan (66%, *n* = 230,421), followed by MRI scan (19%, *n* = 66,333) and PET scan (9%, *n* = 30,722). Scan types like mammography and bone scan are less than 3% of the curated reports. To determine the quality of labeled data, 25% of patient's curated data is verified with a full source document verification (SDV) by a research associate. SDV entails reading over the EMR note and cross checking it with the curated data to verify the information. In this case, the curator read over the impression portion of the radiology note and verified if the curated imaging scan sites, evidence of cancer, status of cancer and other information were correct. For each variable if the information is incorrect or missing, we flag this as an error. Each radiology note is reviewed for an error and each patient can have many radiology notes. Once this SDV was conducted we noted that we had an error rate of 1% for imaging scan site, 2.2% for the presence of cancer, and 1.5% for status of cancer.

**FIGURE 3:**
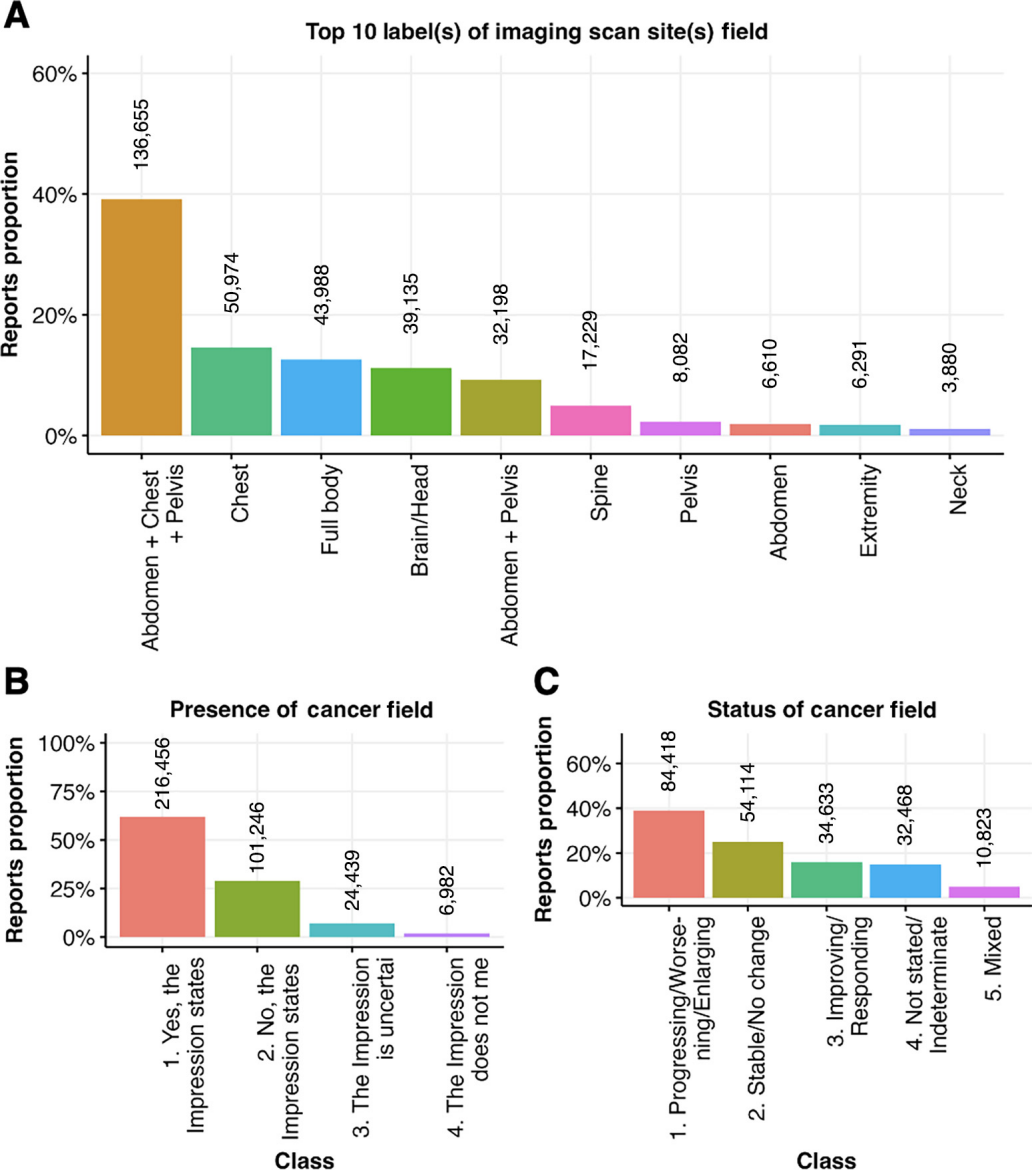
Data summary of curated radiology reports by choices and labels of curation fields in Table 1. **A,** Distribution of reports by top 10 (98% of data) image scan site labels selections for imaging scan site field. **B,** Distribution of reports by class choices for evidence of cancer field. **C,** Distribution of reports by class choices for cancer status field.

For model training, the data is partitioned for training, evaluation and testing while achieving the balance in stratification between the data splits. To identify stratification the task output which is the selected label(s) or class from curation is chosen. Figure 3A shows distribution of the curated reports by top 10 label(s) chosen in prediction of imaging scan site(s) which constitute about 98% of the data. Most of the reports (40%, *n* = 136,655) have multiple labels (abdomen, chest, and pelvis). This label selection is used for stratification of the data to train the model for prediction of imaging scan site. The class distribution for task of predicting presence of cancer is shown in Fig. 3B, where class 1 (“Yes, the Impression states or implies there is evidence of cancer”) is dominant with 62% proportion (*n* = 216,456) and class 4 (“The Impression does not mention cancer”) has lowest proportion 2% (*n* = 6,982). Because of severe skew of class proportions partitioning the data with balanced class proportions is important for training a good model. Likewise, the class distribution for prediction of cancer status is plotted in Fig. 3C, where class 1 (“Progressing/Worsening/Enlarging”) is largest proportion of 39% (*n* = 84,418) and class 5 (“Mixed”) is least proportion 5% (*n* = 10,823).

Three different NLP model classifiers are trained and deployed for each of the tasks using aforementioned methodology. The final selected models are deployed and accessible for inference by API endpoint which supports fast programmatic use of the model. The fine-tuned NLP models are evaluated using a held-out test dataset. The test data is passed as payload in small batches to model APIs. This pipeline achieves a high-throughput of approximately 0.08 seconds per report without SHAP explainability.

For each class or label, we use one versus rest approach (OvR) to compute classifier metrics. Because all three models required different data partition depending on label(s) or class stratification, therefore each model has their own task dependent held-out test data. The models were evaluated by using the threshold of 50% for label and class probabilities because the training was performed in same setting. The results for each of the classifier model are tabulated in Tables 2–4. The exact accuracies of fine-tuned models for tasks – imaging scan site, presence of cancer and cancer status are 99.6% [95% confidence interval (CI), 99.45%–99.7%], 89.2% (95% CI, 88.9%–89.5%), and 85.45% (95% CI, 85.4%–85.5%), respectively. Weighted accuracies of the models are 99.9%, 93.4%, and 93.1%, respectively. Weighted F_1_ scores for the models are 99.4%, 88.8%, and 85.5%, respectively.

**TABLE 2:** Classification metric of the multi-label classifier model to predict imaging scan site using one versus rest approach on held out test data

OvR Classifier evaluation on hold out test data						
Label	Prevalence	Precision	Recall	F_1_ Score	AUCROC	Accuracy
1. Brain/Head	11.4%	99.1%	99.2%	99.2%	99.9%	99.8%
2. Spine	5.0%	98.3%	98.6%	98.5%	99.7%	99.8%
3. Neck	1.3%	89.9%	93.8%	91.8%	98.7%	99.8%
4. Chest	54.6%	99.4%	99.5%	99.4%	99.8%	99.4%
5. Abdomen	51.1%	99.3%	99.7%	99.5%	99.9%	99.5%
6. Pelvis	50.9%	99.1%	99.4%	99.3%	99.8%	99.2%
7. Extremity	2.0%	94.5%	95.6%	95.1%	99.5%	99.8%
8. Full body	12.6%	99.2%	99.7%	99.5%	100.0%	99.9%
Weighted average		99.4%	99.4%	99.3%	99.4%	99.9%
OvR Classifier evaluation on external test data: MIMIC-CXR						
4. Chest	100%	100%	96%	97.8%	NA	96%

**TABLE 3:** Classification metric of the multi-class classifier model to predict presence of cancer using one versus rest approach on held out test data

OvR Classifier evaluation on hold out test data						
Class	Prevalence	Precision	Recall	F_1_ score	AUCROC	Accuracy
1. Yes, the Impression states or implies there is evidence of cancer	62.2%	93.4%	96.1%	94.8%	98.3%	93.4%
2. No, the Impression states or implies there is no evidence of cancer	29.0%	87.1%	86.8%	86.9%	97.5%	92.4%
3. The Impression is uncertain, indeterminate, or equivocal	6.8%	74.1%	49.2%	59.1%	93.5%	95.3%
4. The Impression does not mention cancer	2.0%	62.5%	34.4%	44.4%	97.6%	98.3%
**Weighted average**		88.8%	89.2%	88.8%	97.6%	93.4%

**TABLE 4:** Classification metric of the multiclass classifier model to predict cancer status using one versus rest approach on held out test data

OvR Classifier evaluation on hold out test data						
Class	Prevalence	Precision	Recall	F_1_ score	AUCROC	Accuracy
1. Progressing/Worsening/Enlarging	38.9%	88.7%	87.0%	87.9%	96.5%	90.7%
2. Stable/No change	24.7%	89.9%	88.2%	89.1%	98.3%	94.6%
3. Improving/Responding	15.9%	87.6%	90.7%	89.1%	98.7%	96.5%
4. Not stated/Indeterminate	15.7%	77.0%	72.6%	74.7%	94.4%	92.3%
5. Mixed	4.8%	68.1%	74.4%	71.1%	97.9%	97.1%
**Weighted average**		85.6%	85.5%	85.5%	97.0%	93.1%

The model to predict image scan sites is very close to a perfect classifier. The binary classification metrics for each scan site is given in Table 2, all individual labels have high performing results. This high accuracy in prediction of imaging scan site(s) are identifiably present in beginning part of the radiology reports (see example in Fig. 2C). To examine generalizability of this model, we evaluate the deployed model on external dataset that includes only chest X-ray reports (32). The model achieves 96% (95% CI, 94.6%–97.4%), accuracy in identifying “chest” label in MIMIC-CXR radiology reports (see bottom of Table 2).

Evaluation of the multiclass classifier model to predict presence of cancer has F_1_ score of 89%, and a high F_1_ score 95% for the class 1 (“Yes, the Impression states or implies there is evidence of cancer”) that has high prevalence 62.2% (see Table 3). Hence the model has high precision and recall in reports that contains evidence of cancer. Reports corresponding to this class which indicates presence of cancer are then used to predict cancer status from third fine-tuned NLP model. Class 3 (“The Impression is uncertain, indeterminate, or equivocal”) and class 4 (“The Impression does not mention cancer”) have lower F_1_ scores 49.2% and 34.4%, respectively. This could be due to lower prevalence in the data, 6.8% and 2%, respectively.

The model which predicts the cancer status shows combined F_1_ score close to 90% for first three classes which have combined prevalence of 80% (refer Table 4). The rest of the class refer to ambiguous or mixed cases where the model accuracy is lower. Class 4 (“Not stated/Indeterminate”) has prevalence of 15.7% but lower F_1_ score (74.7%) than Class 3 (“Improving/Responding”) which has similar prevalence of 15.9% and higher F_1_ score (89.1%). We suspect this is due to nature of reports itself which lack definitive information regarding the cancer status and perhaps the human curators face similar uncertainty in annotating this class.

Model's performance is linked to probability threshold for class or label selection. This probability threshold can be used as a knob to trade-off between precision and recall. In our deployed pipeline, we choose 50% as the threshold which sits very close to threshold values which maximize F_1_ score. Other methods of choosing threshold values attempt to minimize appropriate risk measures.

## Discussion

**FIGURE 4:**
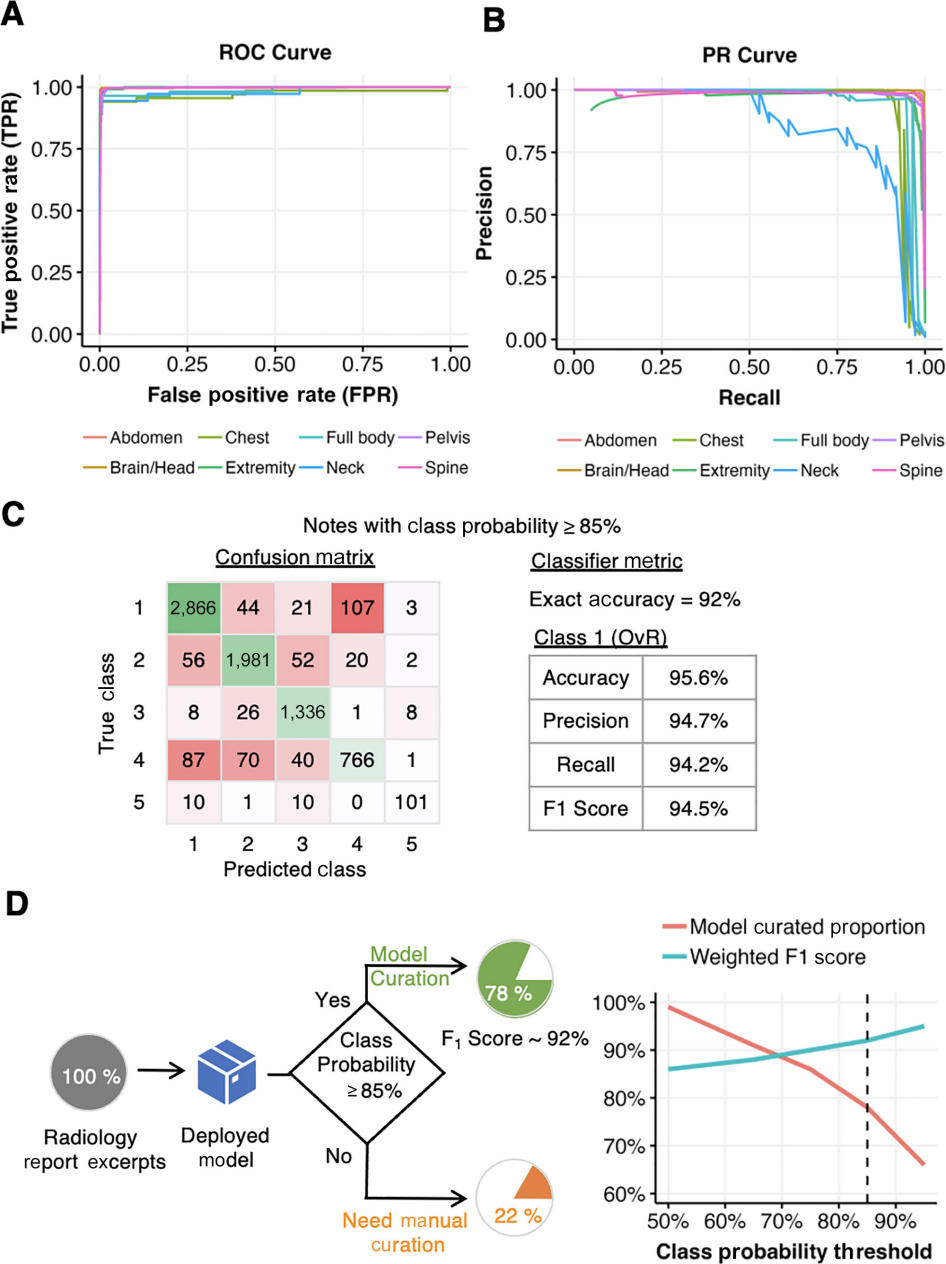
Model evaluation on held-out data and model-assisted curation workflow. **A,** ROC curve of model to predict imaging scan site on held data with MRI scans only. **B,** PR curve of model to predict imaging scan site on held data with MRI scans only. **C,** Confusion matrix and classifier metrics of model output of prediction of cancer status with class probabilities exceeding threshold value of 85% (*n* = 7,617). **D,** Model-assisted curation workflow for prediction of cancer status with threshold 0.85. Approximately 80% of reports exceed threshold of 0.85 which model curates with 92% F_1_ score.

The model to predict imaging scan sites are highly accurate and lends to the possibility of complete replacement of the manual curation of this field. It is also likely that models with smaller number of parameters and hence lower memory footprint could perform equally well. The report structure depends on the type of scan (e.g., CT, PET, MRI, mammography). The model test score is equally high for all scan types. Receiver operating characteristic (ROC) and precision-recall (PR) curves for MRI scans are shown in Fig. 4A and B, respectively. Because of very low prevalence (<1%) and hence very high imbalance of true and negative classes for the label “Neck”, its F_1_ score is lower than other labels in PR curve.

Although, the model to predict the presence of cancer has high F_1_ score and accuracy on dominant classes 1 and 2 (both weighted are ∼93%), but the other two classes have high accuracy and low F_1_ score (see Table 3). Despite lower prevalence, classes 3 and 4 comprise of reports which have no clear evidence of cancer in the impression. A detailed textual analysis is needed on these classes to determine if these classes contain ambiguous or no evidence. The calibration of model's accuracy to manual curation accuracy can also shed light to the poor performance of model on these classes. Similar interpretation can be made on low performance of model to predict cancer status on classes 4 and 5 (see Table 4).

The label or class probabilities from model's output can be used as a gauge in confidence of the model's output. If the model is evaluated as a classifier by considering reports for which the predicted class or label probability (maximum value for either true or false categories), then we find the model tend to perform better. Figure 4C shows confusion matrix of class predictions of cancer status by considering reports with class probabilities more than 85% for metrics evaluation. The confusion matrix tabulates the count of reports with true classes against their respective predicted classes. In this case the exact accuracy of the model improved from 85.45% to 93%, the classifier metrics for class 1 (OvR) presented in Fig. 4C also show marked improved in F_1_ score to 94.5% (from 87.9% in Table 4). The proportion of reports which exceed 85% class probabilities about 78% in the hold out dataset. This implies a large burden of manual curation of radiology reports can be automated with model's prediction. An example of a proposed model assisted curation workflow with prediction of cancer status is shown in Fig. 4D. The reports for which model's prediction is below threshold will be manually curated. All the reports with high model probability prediction can be curated using model's output. A small portion of these reports may be manually curated to monitor the model's performance which could change due to drift in the data. In the same figure, the plot of weighted F_1_ score of all classes and proportion of reports eligible for model curation shows the trade-off between model performance and extent of automation of curation process. It is evident that models which are comparable to human performance can help automate large portion of radiology report curation with higher overall accuracy and F_1_ score. This implementation will result in reducing curation related to expense and hence speed up reports annotation process delivering structured radiology data of patients with cancer.

Our implementation ensures transparency and explainability of the model. Procedure of fine-tuning and deployment of the models as API services guarantees privacy and ethical use of AI. This work is implemented amidst the ongoing manual curation process. Hence the retrospective data used to train these models may not represent true data distribution of the radiology reports in MSKCC. With progressing manual curation, the models are being evaluated and updated as necessary. Complete calibration of model's output with curators’ performance is also under progress. The proposed model assisted curation workflow incorporates continuous monitoring of the model.

Because the origin of the radiology reports is at MSKCC, the trained NLP may not generalize well on radiology reports from external healthcare centers. Because of lack of external dataset curated in same methodology as ours it is difficult to evaluate model's generalizability. However, a high accuracy of imaging scan site on reports in MIMIC-CXR (32) is encouraging (see Table 2).

Current evolution of LLM's is fast paced with emergence of cheaper and faster models. The tasks in this study are discriminative in nature where BERT models have comparable performance with LLMs (28), but newer LLMs or improved prompting techniques and workflows such as retrieval augmented generation (RAG) need to be explored in further study. Unlike LLMs, the BERT based models’ predictions are from closed domain and hence do not suffer from hallucination. Because of limited size of input sequence in BERT models we have used relevant excerpt of the radiology reports. Recent LLMs have much larger input context window can consume the whole report.

Accurate and structured data is indispensable for cancer research. Although manual curation is an established gold standard method for extracting cancer data model elements from radiology reports, artificial intelligence can improve the curation process in multiple facets. There are incentives to use trained language models in conjunction with human curators. Our work successfully demonstrates use of NLP model's assistance for fast and accurate curation of reports. This methodology of using AI to assist in extraction of clinically relevant terms in radiology can be easily extended to other clinical and bio-medical domains. Leveraging AI to improve quality of data will have meaningful impact in cancer research.
